# Prof. Charles E. Dunn.

**Published:** 1868-05

**Authors:** 


*Prof. Charles E. Dunn.*—We are pleased to see among the Faculty of the University Dispensary School of Practical Medicine and Surgery, Louisville, Kentucky, the name of Chas. E. Dunn, D.D.S., a graduate of the Baltimore College of Dental Surgery of the class of 1860, as Professor of the Department of Dental Surgery.

The Summer Session of this excellent school commenced on the 1st Monday of April last.

